# Diagnosing preclinical and clinical Alzheimer's disease with visual atrophy scales in the clinical practice

**DOI:** 10.1055/s-0045-1802960

**Published:** 2025-02-28

**Authors:** Karen Luiza Ramos Socher, Douglas Mendes Nunes, Deborah Cristina P. Lopes, Artur Martins Novaes Coutinho, Daniele de Paula Faria, Paula Squarzoni, Geraldo Busatto Filho, Carlos Alberto Buchpiguel, Ricardo Nitrini, Sonia Maria Dozzi Brucki

**Affiliations:** 1 Universidade de São Paulo, Faculdade de Medicina, Departamento de Neurologia, Grupo de Neurologia Cognitiva e do Comportamento, São Paulo SP, Brazil.; 2 Universidade de São Paulo, Faculdade de Medicina, Instituto de Radiologia, Unidade de Neurorradiologia, São Paulo SP, Brazil.; 3 Universidade de São Paulo, Faculdade de Medicina, Hospital das Clínicas, Departamento de Radiologia e Oncologia, Laboratório de Medicina Nuclear (LIM/43), São Paulo SP, Brazil.; 4 Universidade de São Paulo, Faculdade de Medicina, Departamento de Psiquiatria, São Paulo SP, Brazil

**Keywords:** Alzheimer Disease, Magnetic Resonance Imaging, Atrophy

## Abstract

**Background** Visual atrophy scales from the medial temporal region are auxiliary biomarkers of neurodegeneration in Alzheimer's disease (AD). Therefore, they may correlate with progression from cognitively unimpaired (CU) status to mild cognitive impairment (MCI) and AD, and they become a valuable tool for diagnostic accuracy.

**Objective** To compare the medial temporal lobe atrophy (MTA) and entorhinal cortex atrophy (ERICA) scores measured through magnetic resonance image (MRI) scans as a useful method for probable AD diagnosis regarding clinical diagnosis and amyloid positron-emission tomography (PET).

**Methods** Two neurologists blinded to the diagnoses classified 113 older adults (age > 65 years) through the MTA and ERICA scores. We investigated the correlations involving these scores and sociodemographic data, amyloid brain cortical burden measured through PET imaging with (11)C-labeled Pittsburgh Compound-B (11C-PIB PET), and clinical cognitive status, in individuals diagnosed as CU (CU; *N* = 30), presenting mild cognitive impairment (MCI, *N* = 52), and AD patients ( *N* = 31).

**Results** The inter-rater reliability of the atrophy scales was excellent (0.8–1) according to the Cohen analysis. The CU group presented lower MTA scores (median value: 0) than ERICA (median value: 1) scores in both hemispheres. The 11C-PIB-PET was positive in 45% of the sample. In the MCI and AD groups, the ERICA score presented greater sensitivity, and the MTA score presented greater specificity. The accuracy of the clinical diagnosis was sufficient and no more than 70% for both scores in AD.

**Conclusion** In the present study, we found moderate sensitivity for the ERICA score, which could be a better screening tool than the MTA score for the diagnosis of AD or MCI. However, none of the scores were useful imaging biomarkers in preclinical AD.

## INTRODUCTION

According to the World Health Organization (WHO), currently there are 50 million people with dementia, and several live in low- and middle-income countries with an expectation of higher rates of increase in prevalence in underdeveloped and developing countries in the next years compared with the estimates for the developed world. In Brazil, the prevalence of dementia ranges from 5.1 to 17.5%. 1 2 3 An epidemiological study on dementia in the Brazilian population older than 65 years reported Alzheimer's disease (AD) in 55.1% of the cases. 4 Regarding mild cognitive impairment (MCI) or cognitive impairment, no dementia (CIND), the prevalence ranges from 6.1 to 19.5%, with an incidence of 13.2/thousand person-years in community studies. 3 5 6

Recent guidelines 7 integrate clinical and pathology as amyloid, tau, and neurodegeneration (ATN) to diagnose AD with amyloid-β as a core criteria, and added imaging biomarkers such as positron emission tomography with (11)C-labeled Pittsburgh Compound-B (11C-PIB PET), and Tau -protein PET imaging, to the clinical criteria to improve the reliability of the early clinical diagnosis of AD. But in most low- and middle-income countries, the availability of these PET biomarkers is limited, mainly due to their high cost and to logistics, so the search for other potential imaging biomarkers with higher availability and diagnostic accuracy is fully justified in this context. 7 8 Often, only cranial magnetic resonance imaging (MRI) is available to assess the severity, progression of symptoms, and biological disease by identifying amyloid pathology, atrophy, and degeneration. 9 Atrophy scales from the hippocampal formation are used to aid in the diagnosis of possible AD. 10 11

In 1992, Scheltens et al. 10 described and validated a visual assessment of medial temporal lobe atrophy (MTA) on MRI and distinguished patients with AD from those with normal cognitive status, with the main purpose of differentiating normal aging atrophy from pathological changes. In 2016, Harper et al. 8 compared the visual classification scores in the diagnosis of dementia; MTA was positively associated with loss of hippocampus volume and provided a reliable score to identify late-onset AD. In 2018, Enkirch et al. 11 established another visual score focused on entorhinal cortex atrophy (ERICA) and atrophy of the transentorhinal region, as they are usually the first brain structures affected in AD, indicating a potentially higher diagnostic accuracy of the ERICA score compared to the MTA score.

The present study aims to compare the specificity and sensitivity of the MTA and ERICA scores on MRI as potential neurodegenerative imaging biomarker tools to help in the continuum clinical diagnosis of AD, from pre-clinical to clinical symptoms, in a context in which there is no access to the amyloid pathological signature of 11C-PIB PET.

## METHODS

### Participants

**Figure 1:**
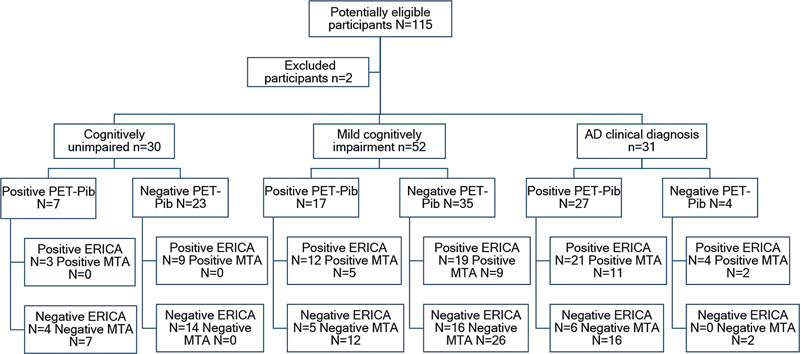
Flowchart and classification according to the entorhinal cortex atrophy (ERICA) and medial temporal lobe atrophy (MTA) scores.

The current retrospective study included 115 individuals who had been followed by the Brazilian Aging and Memory Research Outpatient Clinic from the Division of Neurology and Psychiatry of Hospital das Clínicas da Faculdade de Medicina da Universidade de São Paulo (HCFMUSP) and had undergone MRI and 11C-PIB PET within the maximum interval of 6 months from 2014 to 2017 ( Figure 1 ). They were classified as cognitively unimpaired (CU), mild cognitive impairment (MCI), and AD patients based functional clinical diagnosis by analyzing their records of neuropsychological tests and instrumental activities of daily living according to the Jak/Bondi and Petersen/Winblad criteria (functional activities questionnaire). 12 13 14 15

The medical records and baseline images were reviewed for data on sex, age, level of schooling, clinical diagnosis, and after the classification of the MRI images and 11C-PIB PET.

The subjects were classified as positive or negative 11C-PIB-PET according to the framework previously described in Bussato et al. 16 The images were rated as positive if there was an increase in uptake in the cortical gray matter (GM) causing a loss of GM to white matter (WM) contrast, in at least two of the six following areas: frontal, temporal, lateral parietal, precuneus, anterior cingulate, and posterior cingulate cortices, or if only a single large GM cortical area presented a strong diffuse uptake of the tracer. The images were rated as negative when there was a clear separation between GM and WM, with strong WM uptake and no significant GM uptake. 17 18 19 20

All patients or caregivers provided informed consent in consonance with the Declaration of Helsinki, and the study was approved by the Ethical Committee of the Review board of HCFMUSP (under CAPPesq number 368.037).

### Magnetic resonance imaging acquisition and classification

All the MRI scans were acquired using 3 scanners (Achieva 3T, Philips Healthcare, Best, Netherlands; Excite HDX 1,5T, General Electric Healthcare, Chicago, IL, United States; and Magnetom Spree 1,5T, Siemens Medical, Erlangen, Germany) using the same acquisition and processing parameters. Coronal sections aligned to the hippocampal angulation with 1 mm in thickness were evaluated after software (Intellispace PACS Radiology, Version 4.4.541.1, Philips Healthcare) multiplanar reconstruction of a high-spatial-resolution three-dimensional T1-weighted sequence (isotropic three-dimensional gradient-echo; voxel size = 1.0 × 1.0 × 1.0 mm; repetition time [RT] = 6.5 ms, echo time [ET] = 2.9 ms; field of view [FOV] = 256 mm; flip angle = 9°). The total acquisition time was of 5 m and 30 s. 11

**Figure 2:**
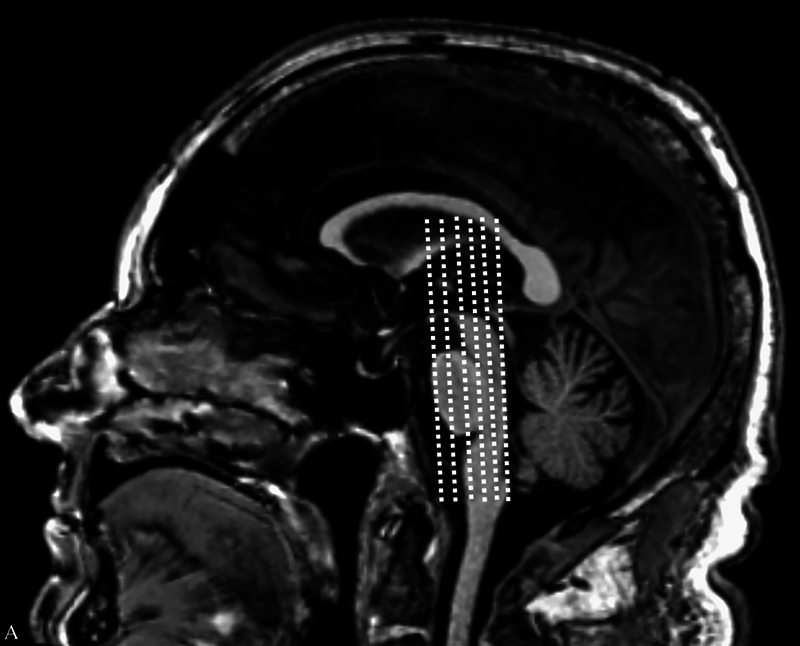
Evaluation of T1-weighted images in multiplayer reconstruction, from the level of the uncus-amygdala complex anteriorly to the dorsal hippocampus posteriorly, at the level of the mammillary bodies.

The T1-weighted images were evaluated for the classification in multiplayer reconstruction from the level of the uncus-amygdala complex anteriorly to the dorsal hippocampus posteriorly, at the level of the mammillary bodies ( Figure 2 ). 11

The MRI scans of all patients were reviewed by two independent cognitive neurologists trained by one expert neuroradiologist (with 10 years of experience) to assess the MTA and ERICA scores. The raters were blinded to the clinical diagnoses, 11C-PIB PET status, and any scores determined by other raters.

**Figure 3:**
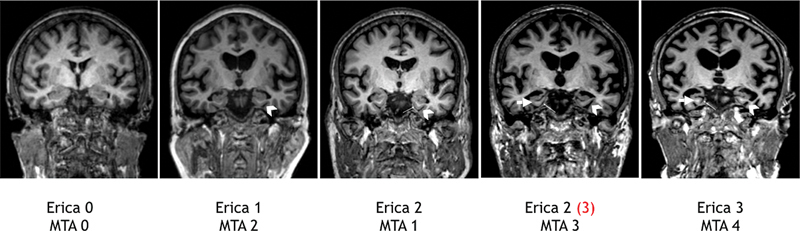
Image example of ERICA and MTA scores for both hemispheres. Note that the MTA score of 3 in the left hemisphere corresponds to an ERICA score of 3 (highlighted in red), while the right hemisphere has an ERICA score of 2 (indicated in black).Positive ERICA scores (2 points) are for patients under 75 years old.

The MTA score comprises the evaluation of the highest vertical hippocampal formation, the hippocampus itself and the subiculum, the para-hippocampus, the greater vertical width of the choroidal fissure and temporal horn width to determine the score and adjust to age. The score ranges from 0 to 4: a score ≥ 2 in subjects younger than 75 years, and a score ≥ 3 in people aged ≥ 75 years indicates probable AD ( Figure 3 ). 10

The ERICA score comprises the evaluation of the entorhinal cortex at the level of the mammillary bodies, the volume of the entorhinal cortex and parahippocampal gyrus, the widening of the collateral sulcus, the atrophy with the detachment of the entorhinal cortex from the cerebellar tentorium, called “tentorial cleft sign,” and the marked atrophy of the parahippocampal gyrus with a large gap between the entorhinal cortex and the cerebellar tentorium. The score ranges from 0 to 3, and a score ≥ 2 indicates probable AD, with high diagnostic accuracy ( Figure 3 ). 11

Both scores were used to analyze the right and left hemispheres simultaneously. If there was any variation between the scores, the higher value was selected for the subsequent analysis. A score was considered positive even if it appeared in just one hemisphere.

### Statistical analysis

The statistical analysis was performed using the IBM SPSS Statistics for Windows (IBM Corp., Armonk, NY, United States) software, version 27.0. The agreement between raters in the analysis of the ERICA and MTA scores was assessed through the Cohen's Kappa (κ) test. The specificity, sensitivity, accuracy, negative predictive value (NPV) and positive predictive value (PPV) were calculated according to 11C-PIB-PET and divided by clinical diagnosis. The Chi-squared test was used to analyze the 11C-PIB-PET status, sex, and clinical diagnosis, and the Kruskal-Wallis test for independent samples was used to compare the age and level of schooling of the clinical groups, as well as the visual scores.

## RESULTS

We selected 115 patients, who were classified as AD, MCI, and CU by clinical diagnosis, and their MRI scans were reviewed and classified according to the MTA and ERICA scores. Two patients were excluded from this cohort due to corrupted MRI files ( Figure 1 ), so the final sample was composed of 113 subjects. The sample had a predominance of females, median of 11 years of schooling, and median age of 73 years. Table 1 shows the demographic and clinical characteristics and the visual scores.

**Table 1:** Demographics and MTA and ERICA scores according to the diagnostic group

		CU ( *n* = 30)	MCI ( *n* = 52)	AD ( *n* = 31)	*p* -value
Age (in years): median (IQR)		71 (60–81)	73.211 (61–86)	76 (60–87)	*0.178
Sex: n (%)	Female	24 (21.20%)	40 (35.40%)	20 (35.40%)	^#^ 0.324
	Male	6 (5.30%)	12 (10.60%)	11(9.70%)	
Years of schooling: median (IQR)		11 (4–20)	10 (2–19)	11 (2–20)	*0.101
Amyloid PET: n (%)	Positive ( *n* = 51)	7 (6.20%)	17 (15%)	27 (23.90%)	^#^ 0.00001
	Negative ( *n* = 62)	23 (20.40%)	35 (31%)	4 (3.50%)	
ERICA – right hemisphere: mean(± SD)	Rater 1	1(± 0.99)	1.5(± 1.09)	2(± 0.81)	*0.004
	Rater 2	1(± 0.94)	1,5(± 1.03)	2(± 0.89)	*0.009
ERICA – left hemisphere: mean(± SD)	Rater 1	1(± 0.96)	1,5(± 1.01)	2(± 1)	*0.00001
	Rater 2	1(± 0.96)	1,5(± 0.93)	2(± 0.98)	*0.001
MTA – right hemisphere: mean(± SD)	Rater 1	0(± 0.89)	1(± 1.28)	1(± 1.46)	*0.00001
	Rater 2	0(± 0.89)	1(± 1.23)	1(± 1.49)	*0.00001
MTA – left hemisphere: mean(± SD)	Rater 1	0(± 0.86)	1(± 1.09)	1(± 1.36)	*0.002
	Rater 2	0(± 0.89)	1(± 1.08)	1(± 1.34)	*0.002

Abbreviations: AD, Alzheimer's disease; CU, cognitively unimpaired; ERICA, entorhinal cortex atrophy; IQR, interquartile range; MCI, mild cognitive impairment; MTA, medial temporal lobe atrophy; PET, positron-emission tomography; SD, standard deviation.

Notes: *Kruskal-Wallis test; ^#^ Chi-squared test.

According to the clinical diagnosis, we observed different positivity rates in the 11C-PIB PET scans of AD patients (87%; 27/31), MCI patients (32%; 17/52; 46 amnestic and 6 non-amnestic), and CU subjects (23%; 7/30).

The visual scales presented good to excellent interrater reliability according to the Cohen's κ analysis, of 0.8 to 1 in the CU group, 0.8 in the MCI group, and 0.8 to 0.9 in the AD group. There was no difference in the assessment regarding brain hemispheres.

**Figure 4:**
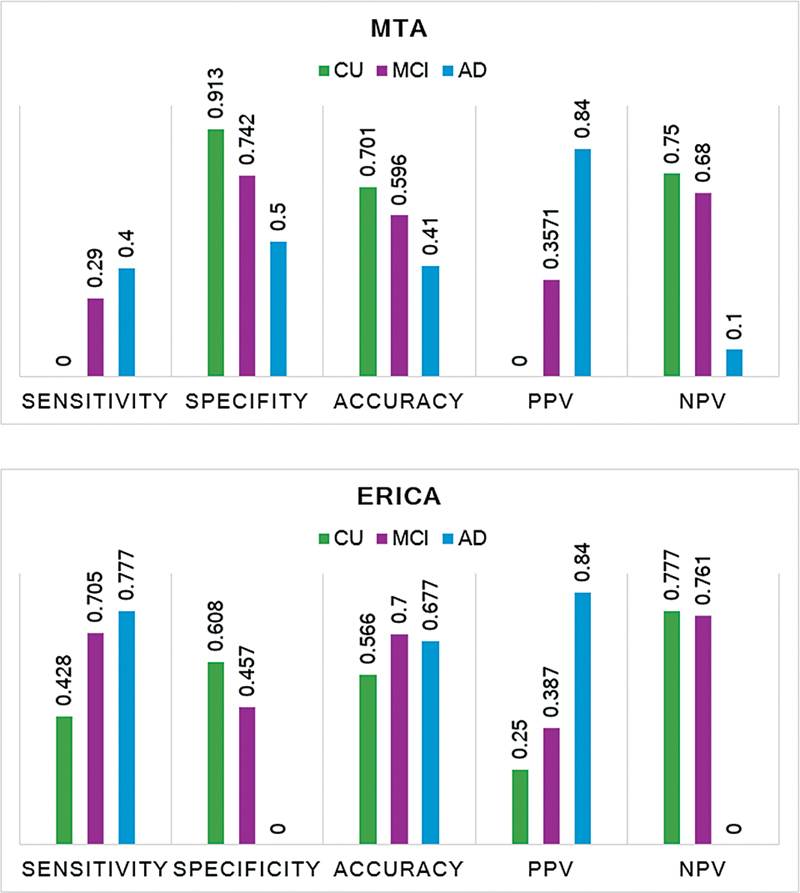
Sensitivity, specificity, accuracy, positive predictive value (PPV), and negative predictive value (NPV) of the ERICA and MTA scores according to clinical diagnosis.

The AD patients presented a median ERICA score of 2 and a median MTA score of 1 for both hemispheres compared to groups without dementia ( *p* < 0.01) ( Table 1 ). The sensitivity of the ERICA score in the AD group according to 11C-PIB-PET status was of 77.7%, and that of the MTA score, of 40.7%; the specificity of the MTA score was 50% to clinical diagnosis. There were no true negatives in our sample to validate the specificity of the ERICA score; the PPV was of 84% for the ERICA and MTA scores ( Figure 4 ). Analyzing only the 27 positive 11C-PIB PET, 10 (37.3%) of them presented a positive ERICA score and a negative MTA score, 11 (40.7%) presented positive scores on both, and 6 (22%), negative scores.

The MCI group presented a median ERICA score of 1.5 and a median MTA score of 1 for both hemispheres according to the 11C-PIB PET status ( Table 1 ). The sensitivity rates for the ERICA and MTA scores were of 70.5% and 29%, respectively; the specificity was of 45.7% for the ERICA score, and of 74.2% for the MTA score. The NPV was higher for the ERICA score, and the PPV was similar for both scores ( Figure 4 ). Reviewing only the 17 positive 11C-PIB PET scans, 7 (41%) of them presented positive ERICA scores and negative MTA scores, 5 (29%) presented positive scores on both, and 5 (29%) presented negative scores on both.

The CU group presented a median ERICA score of 1 and a median MTA score of 0 for both hemispheres according to the 11C-PIB PET status ( Table 1 ); the sensitivity of the ERICA score in this group was of 40%. However, it was not possible to determine the sensitivity of the MTA score due to our sample size. The specificity of the ERICA score was of 60% and that of the MTA score, of 90%. The PPV was similar for both scores: 77% for the ERICA and 75% for the MTA ( Figure 4 ). By reviewing only the 6 positive 11C-PIB PET scans in the CU group, 7 (33%) of them presented positive ERICA scores and negative MTA scores, and 4 (66%) presented negative scores on both.

Analyzing preclinical dementia by combining the CU and MCI groups with 11C-PIB PET status, the sensitivity was of 30% and the specificity was of 70% for both scores. The NPV was higher for the MTA score (80%), and the PPV was of 62% for the ERICA, and of 20% for the MTA. By reviewing only the 23 positive 11C-PIB PET scans, 9 (39.1%) presented a positive ERICA score and a negative MTA, 5 (21.4%) presented positive scores on both, and 9 (39.1%) presented negative scores on both.

The accuracy of the ERICA score in terms of the 11C-PIB PET status was of 56.6% in the CU group, of 53.8% in the MCI group, and of 67.7% in the AD group; as for the MTA score, the accuracy was of 70%, 59.6%, and 41.9%, respectively. When analyzed in terms of preclinical dementia, the accuracy was of 54% for the ERICA, and of 63.4% for the MTA.

## DISCUSSION

We observed modest accuracy for both visual scales for the diagnosis of AD, with better rates in the dementia group. Both scores presented excellent inter-rater agreement, proving to be useful tools in the clinical practice, in settings where other biomarkers are unavailable. In 2004, Klunk et al. 21 published the first study in humans using amyloid PET, and a strong correlation between 11C-PIB PEPT image uptake, amyloid deposition observed in pathology studies, and the clinical diagnosis of AD excluded. 21 22 However, despite being an excellent diagnostic tool, access to this exam is challenging in low- and middle-income countries, mainly due to the high cost. Thus, the visual analysis of cortical atrophy using MRI certainly could be more accessible and affordable to diagnose AD in developing countries. 23 We analyzed through a clinical progression, from asymptomatic to dementia, if the ERICA and MTA scores are promising biomarkers compared with AD's pathological findings using amyloid measured by 11C-PIB PET. 10 11 16

The ERICA score presented a sensitivity of 83% and a specificity of 93% in the study by Enrich et al., 11 with an accuracy of 91% for the MTA score by Schelten et al. 10 , which reported a sensitivity of 81% (mean age of the patients: 72.8 years) and a specificity of 67% (mean age of the patients 70.9 years) for the diagnosis of AD. Wei et al. 24 have suggested changing the cut-off values for the MTA score according to age groups to improve the outcomes. Subjects under 65 years of age should have positive scores ≥ 1, with a sensitivity of 92.3% and specificity of 84.5%, those between 65 and 74 years of age should have positive scores ≥ 1.5, with a sensitivity of 90.4% and a specificity of 85.2%, and those over 75 years of age should have positive scores ≥ 2, with a sensitivity of 70.8% and a specificity of 82.3% for the diagnosis of AD. 24

In the present analysis, the ERICA score presented a sensitivity of 77.7% in the AD group, which was similar to the rate reported by Enkirch SJ et al. ^11^ The accuracy found in the current study was lower than expected, of 67.7%. The MTA presented the worst values in the AD group, with a sensitivity and accuracy of 40%. However, our MTA analysis (mean age of the patients: 76 years) probably reached lower values due to the correlation of a pathological biomarker, 11C-PIB-PET, with several negative cases. This may be explained by the fact that 10 to 30% of the individuals clinically diagnosed with AD dementia did not present neuropathological changes on autopsy, nor positive 11C-PIB-PET. Therefore, the ERICA visual analysis of atrophy on MRI was preferable when confirming AD. 25 It is also important to consider that our sample of clinical AD dementia diagnosis has 4 cases that were 11C-PIB-PET negative, 13% have more than 80 years of age, and may have been misdiagnosed with AD when they actually probably have LATE pathology. 17 26

The MCI was a very heterogeneous group, with different patterns of clinical presentations; their ERICA and MTA scores were similar, with an accuracy of approximately 50%, and no differences in the visual analysis of cerebral atrophy. The ERICA score presented moderate sensitivity, of 70%, and the MTA score was more specific, at a rate of 74%. Our findings can be explained by a study that evaluated a cortical volume in this group which considered the time of clinical continuum; there were differences regarding the areas of cortical atrophy in the group with early-stage cognitive symptoms in the memory domain, the most affected site would be the mesial temporal lobe, assessed by the MTA score. For those patients diagnosed in the late stage of the MCI, the atrophy area was more significant in the hippocampus and left fusiform similar to those evaluated by the ERICA score. Therefore, we suggest that MCI should be assessed on MRI scans using both visual atrophy scores. 10 11 17

It is also important to make some considerations about our sample and the scores used, which may have had their sensitivity and specificity decreased in borderline clinical stages such as MCI, as they analyze the mesial temporal structures in an oblique angulation and evaluate the intercommissural lines anteriorly and posteriorly, which may justify the underestimation of atrophy in this subgroup. In contrast, the present study suggests that the ERICA and MTA scores may yield better results if the analysis would include the longest perpendicular axis angulation of the hippocampus. 10 11

Unlike the original studies we evaluated the clinical applicability of both scores for asymptomatic individuals, as AD monitoring dementia screening is a common request in the clinical practice. 10 11 Additionally, in the CU group, both scores presented low sensitivity; however, the MTA score presented a greater specificity (of 91%) than the ERICA score (of 60%).

In the clinical practice of a neurologist who has no or limited availability of assessing the amyloid status by different tests and would like to use visual scales of atrophy as a prognostic biomarker as preclinical to clinical diagnosis for AD, our findings showed a moderate sensitivity for the MCI and AD groups, a moderate specificity for the CU and MCI groups, and poor accuracy for both. These findings could be explained by the fact that atrophy is present in advanced stages of the disease, it reflects neurodegeneration, it is not a specific AD biomarker, and it could be related to other types of dementia. 27 According to research on biomarkers, 22 28 neurologists could use these visual tools, mainly the ERICA score, to screen for AD in patients with clinical dementia, but not in those asymptomatic or in the early stages, without dementia.

The limitations of the current study are the relatively small sample from a specialized outpatient clinic, which corresponds to an overestimated visual analysis of areas. Thus, it was important to emphasize that the decrease in the volumetric analysis of the GM of these regions with a different angulation than usual to MRI analysis of the hippocampus could improve our results for screening the clinical spectrum of AD, therefore, this image analysis protocol should be followed to generalize the findings in daily clinical practice. 17 And the most effective screening method is the association of biological and neurodegenerative biomarkers of AD to predict the clinical symptoms and cognitive decline. 29 30

In clinical practice, when neurologists use visual classification scores to diagnose AD and do not have access to other biomarkers, the ERICA score could be a better screening tool for AD diagnosis than the MTA score. However, none of them were useful tools as prognostic biomarkers in preclinical AD.
